# Using automatic speckle tracking imaging to measure diaphragm excursion and predict the outcome of mechanical ventilation weaning

**DOI:** 10.1186/s13054-022-04288-3

**Published:** 2023-01-14

**Authors:** Daozheng Huang, Feier Song, Bangjun Luo, Shouhong Wang, Tiehe Qin, Zhuandi Lin, Tieying Hou, Huan Ma

**Affiliations:** 1 Department of Critical Care Medicine, Guangdong Provincial Geriatrics Institute, Guangdong Provincial People’s Hospital (Guangdong Academy of Medical Sciences), Southern Medical University, Guangzhou, 510080 China; 2 Office of Organ Procurement Organizations, Medical Department, Guangdong Provincial People’s Hospital (Guangdong Academy of Medical Sciences), Southern Medical University, Guangzhou, 510080 Guangdong China; 3 Department of Emergency Medicine, Guangdong Provincial People’s Hospital (Guangdong Academy of Medical Sciences), Southern Medical University, Guangzhou, 510080 China; 4 Department of Critical Care Medicine, Guangzhou Panyu Central Hospital, Guangzhou, 510080 China; 5 Guangdong Clinical Laboratory Center, Guangdong Provincial People’s Hospital (Guangdong Academy of Medical Sciences), Southern Medical University, Guangzhou, 510080 Guangdong China; 6 Medical Department, Guangdong Provincial People’s Hospital (Guangdong Academy of Medical Sciences), Southern Medical University, Guangzhou, 510080 Guangdong China; 7 Department of Cardiology, Guangdong Provincial Cardiovascular Institute, Guangdong Provincial People’s Hospital (Guangdong Academy of Medical Sciences), Southern Medical University, Guangzhou, 510080 China

**Keywords:** Speckle tracking ultrasound, Diaphragmatic excursion, Weaning

## Abstract

**Introduction:**

The speckle tracking ultrasound is an innovative technology enabling distinct assessment of diaphragmatic movement, yet the relative data are scarce. In this pilot study, we sought to evaluate the predictive value of the weaning outcome of automatic speckle tracking in assessing diaphragm excursion.

**Methods:**

This is a prospective, multicenter, observational study. A total of 160 critically ill subjects underwent speckle-tracking ultrasonography of the right/left hemidiaphragm before the spontaneous breathing trial. Meanwhile, the diaphragm excursion and velocity values were measured manually by M-mode ultrasound. Patients were divided into weaning-failure and weaning-success groups. The correlation was assessed between automatic and manual measurement, and the diagnostic efficacy of automatic measured excursion and velocity for predicting weaning outcome was analyzed.

**Results:**

A total of 88 patients completed the follow-up of the weaning outcome. The overall incidence of weaning failure was 43.18%. There was a significant correlation between the automatic measurement of mean excursion and velocity assessed by speckle tracking imaging and manual measurement (R 0.69 and 0.65, respectively). Receiver operating characteristic (ROC) curve analysis showed that the mean excursion and diaphragmatic velocity exhibited high diagnostic values for prolonged weaning [area under the ROC curve (AUROC) 0.824 and 0.786, respectively]. The diaphragmatic excursion showed moderate diagnostic value for predicting both weaning failure and in-hospital death/withdrawal of treatment (AUROC 0.659 and 0.653, respectively).

**Conclusion:**

Automatic speckle tracking analysis of the diaphragm showed high consistency with conventional manual ultrasound measures. Diaphragmatic excursion and its excursion velocity helped predict mechanical ventilation weaning failure, prolonged weaning, as well as in-hospital adverse outcomes, which served as a reliable tool in guiding clinical weaning strategy.

**Key message:**

- Automatic speckle tracking analysis of the diaphragm showed high consistency with conventional manual ultrasound measures.
- Diaphragmatic excursion and its excursion velocity helped predict mechanical ventilation weaning failure, prolonged weaning, as well as in-hospital adverse outcomes.

**Supplementary Information:**

The online version contains supplementary material available at 10.1186/s13054-022-04288-3.

## Introduction

Ultrasound performs static structural analysis as well as dynamic motion to evaluate diaphragm functional changes, allowing direct visualization [1]. Studies have shown that the assessment of diaphragm movement, such as excursion, can help predict the success rate of weaning or prolonged weaning from mechanical ventilation (MV) [2–4]. At present, M-mode ultrasound is commonly used in clinical practice to assess the diaphragmatic movements and diaphragmatic velocity of contraction. It allows more accurate timing of the respiratory cycle, but is worse spatial orientation and is difficult to operate [1]. Also, since there is no real standard for M-line selection, repeatability is poor [5–8]. Speckle tracking ultrasound is an innovative ultrasound technique enabling distinct assessment of muscle function or organ motion, such as velocity, displacement, strain, and strain rate [9, 10]. It tracks the movement of the tissue by tracking the speckle formed by the tissue echo signal on the B image. Through this, the physiological characteristics of the tissue can be quantitatively analyzed. Moreover, via big data training samples, the accurate diaphragm recognition model and speckle tracking algorithm are obtained.

Speckle-tracking echocardiography is a novel and mature technique for assessing myocardial function. Due to the contribution of speckle-tracking echocardiography, we intended to apply this method to diaphragm ultrasound. Despite the structural difference between the diaphragm and myocardium, there is still no readily available product for automatic speckle tracking imaging to measure diaphragmatic parameters. Furthermore, data regarding the relationship between diaphragmatic movement measured by automatic speckle tracking imaging and MV weaning failure in critically ill patients are scarce. In tQ3his pilot study, we sought to evaluate the predictive value for the weaning outcome of automatic speckle tracking in assessing diaphragm excursion and how this compares to manual methods of diaphragm excursion assessment by ultrasound.

## Methods

### Subjects

This prospective, multicenter, observational study was conducted at the intensive care units of Guangdong Geriatric Institute and Guangzhou Panyu Central Hospital, China. All subjects’ families provided written informed consent. The study was performed following the approval of the ethics committee.

Patients were included when they met all of the following criteria: aged ≥ 18 years, received MV for > 48 h, suitable for a spontaneous breathing trial (SBT). The exclusion criteria were as follows: patients with a pre-existent neuromuscular disorder, diaphragmatic paralysis, cervical injury, pneumothorax, or mediastinal emphysema, and if the patient has a poor echogenicity or who were unable to tolerate ultrasound examination.

### Ultrasound imaging and analysis

The measurements of left/right diaphragm excursion and velocity were taken on the M-mode frozen images using the ultrasound machine calibration and algorithm in a supine or semi-recumbent position before SBT by a well-trained expert. All of the patients had spontaneous breathing with the pressure support of 10–12 cm H_2_O. Diaphragm excursion was measured as previously described [11]. TE7 Diagnostic Ultrasound System (C5-2 array probe, Shenzhen Mindray Bio-medical, China) was used. Manual measurement was taken when the patient's breathing was relatively stable and the ultrasound image was steady. Under M-mode ultrasound, the exploration line was selected so that the ultrasound beam was perpendicular to the posterior diaphragm. Meanwhile, the liver, inferior vena cava, and hyperechoic diaphragm line were shown in the plane. The M-mode showed the diaphragm movement along the exploration line. Diaphragm excursion was the vertical distance from the baseline (during exhalation) to the highest point (during inhalation) of the curve. The diaphragmatic velocity of the excursion (cm/s) was calculated as diaphragmatic excursion (cm) divided by the duration of the corresponding excursion (s). An additional file showed this in more detail (Additional file 1).

### Speckle tracking imaging

**Fig. 1:**
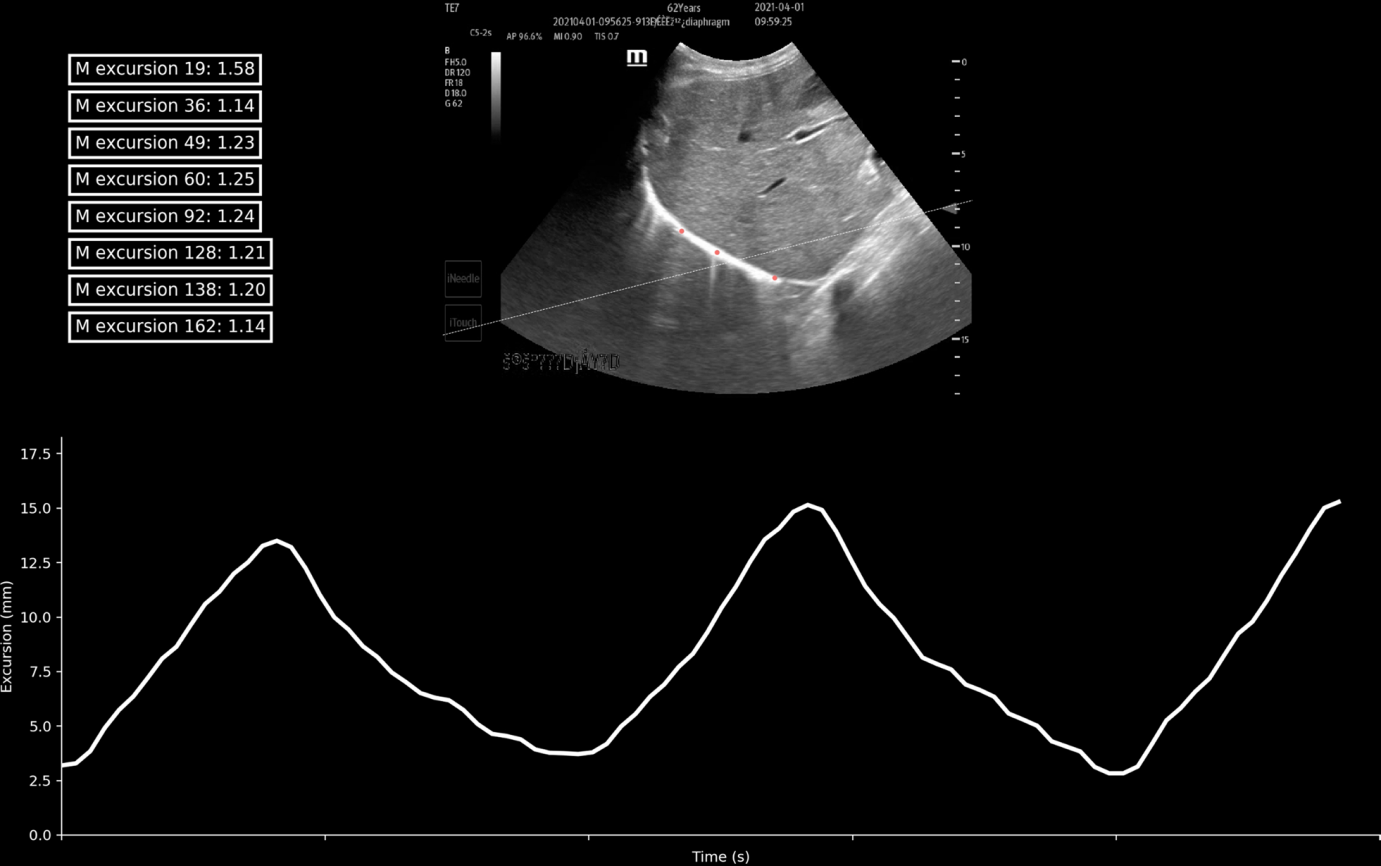
Three regions of interest (ROI) were selected to represent each segment of the diaphragm (color red). The line chart shows that the diaphragm excursion (cm) was measured by one ROI. The anatomic M-line was drawn via calculation of the first excursion (dashed line)

During the identification phase, the videos of the left/right diaphragm motions were saved and diaphragm excursion was detected with the cardiac transducer (SP5-1u probe, Resona7 ultrasound system, Shenzhen Mindray Bio-medical, China) (Additional file 2: Video 1 and Additional file 3: video 2). The automatic speckle tracking imaging was performed using independently developed patent software. During the validation phase, a 15–20-s (to cover 3–5 respiratory cycle) clip was recorded, using TE7 Diagnostic Ultrasound System (C5-2 array probe, Shenzhen Mindray Bio-medical, China). 3 regions of interest (ROI) were traced and the measurements were averaged in the offline analysis. The excursion of the ROI was calculated by the algorithm with the anatomical M-line (Fig. 1) (Additional file 4). Examples of tracking were displayed in the Additional file 8 videos 3 and Additional file 9: video 4. The speckle tracking method for the diaphragm was as follows: (1) the recorded ultrasound data were identified through pattern recognition of deep learning; (2) The region of interest (ROI) was placed to represent each segment of the diaphragm; (3) several candidate anchor points around each ROI were selected to allow adequate tracking; (4) perform matching calculation on the front and back frames of the area where the ROI and the candidate anchor point are located; (5) the point with the highest comprehensive matching calculation coefficient is the movement direction of the ROI; (6) Obtain the motion trajectory of each ROI; 7) calculate the range of motion and thickness change.

### SBT

The criteria for weaning readiness included: (1) respiratory rate ≥ 10 and ≤ 35 breaths per min; (2) PaO_2_/FiO_2_ ratio ≥ 150; (3) positive end-expiratory pressure ≤ 5–8 cm H_2_O; (4) FiO_2_ < 50%; and (5) pH value > 7.25. For patients with chronic obstructive pulmonary disease, the criteria were: (1) pH > 7.30; (2) PaO_2_ > 50 mmHg; and (3) hemodynamic stability with no dynamic changes of myocardial ischemia or hypotension (no vasopressors or little inotropes such as dopamine/dobutamine), in the absence of vasopressors. MV was disconnected from the patient, and an independent source of oxygen was provided through the T-piece. The attempt was targeted at least 30 min and be up to 120 min. SBT was terminated when one of the following signs occurred: (1) rapid shallow breathing index > 105; (2) respiratory rate < 10 and > 35 breaths per min; (3) heart rate > 140 beats per min or changed > 20% compared with the baseline or the new onset of arrhythmia; (4) tidal volume < 4 mL/kg; and (5) SaO_2_ < 90%.

### Outcomes

The weaning outcome was diagnosed successfully if the patient could maintain spontaneous breathing for ≥ 48 h with no need for any level of ventilator support after extubating. Otherwise, the outcome was defined as a weaning failure. Patients were divided into weaning-failure and weaning-success groups. Another classification of patients according to the weaning process includes weaning time. Prolonged weaning was defined as more than 3 times SBT failures or failure to wean within 7 days after the first SBT. Factors that are known to affect weaning outcomes were noted, such as the underlying diseases, ventilation time, weaning time, and relevant blood biochemistry findings.

### Statistical analysis

Pearson’s correlation coefficient was used to assess the correlation between automatic and manual measurement of excursion and velocity. Averaged data were expressed as mean ± standard deviation for continuous variables and as absolute or relative frequencies for categorical variables. An independent sample *t*-test was used to compare continuous variables and the chi-square test or Fisher’s exact tests were used for categorical variables. A Bland–Altman plot was used to describe the agreement between manual and automatic measurement. Receiver operating characteristic (ROC) curve analysis was performed to determine the diagnostic efficacy of automatic measured excursion and velocity for predicting weaning outcomes. The sensitivity and specificity of automatic speckle tracking imaging in predicting the outcome of weaning were calculated and compared with that of manual measurement. All data were handled in R version 4.0.2 and *P* < 0.05 was considered significant and all probability values were 2-sided.

## Results

A total of 160 subjects underwent speckle-tracking ultrasonography between August 2020 and July 2021. Images were captured and visualized with all 160 subjects’ right/left hemidiaphragm before SBT. Meanwhile, the diaphragm excursion and velocity values were measured manually by M-mode ultrasound. Finally, 88 patients completed the follow-up of the weaning outcome and were enrolled in the diagnostic analysis of ultrasonic imaging assessment. Among these patients, the overall incidence of weaning failure was 43.18% (38/88). Of the 88 remaining patients, the overall mean age was 73 ± 14 years, and 59 (69.41%) were male. The mean body mass index was 22.8 kg/m^2^. Demographic factors, comorbidity, respiratory parameter, and laboratory findings did not differ significantly between the weaning-failure group and weaning-success group, except for body mass index, tidal volume, calcium concentration, albumin, hemoglobin, age, and Sequential Organ Failure Assessment score. Pre-SBT baseline characteristics of the subjects by weaning outcome are reported in Table 1.

**Table 1:** Baseline characteristics between weaning-failure and weaning-success groups

	Weaning-success *N* = 50	Weaning-failure *N* = 38	*p* value
Male, *n* (%)	35, 72.9%	24, 64.9%	0.4244
Age, years	70 ± 14	77 ± 13	0.0195
Height, cm	161.2 ± 5.8	163.5 ± 6.9	0.0999
Weight, kg	59.7 ± 6.5	57.2 ± 6.1	0.0711
Body mass index	23.0 ± 2.8	21.6 ± 3.3	0.0273
Temperature, ℃	36.9 ± 0.5	37.7 ± 3.8	0.1455
Systolic blood pressure, mmHg	107 ± 40	103 ± 38	0.6510
Diastolic blood pressure, mmHg	63 ± 26	57 ± 21	0.2501
Heart rate, beats per minute	107 ± 25	107 ± 31	0.9455
Respiratory rate, breaths per minute	24 ± 8	25 ± 9	0.7609
Tidal volume, ml	349.0 ± 37.1	373.2 ± 36.9	0.0032
PEEP, cmH_2_O	4.7 ± 1.2	4.6 ± 1.2	0.5825
pH	7.3 ± 0.1	7.3 ± 0.2	0.1374
PaO_2_, mmHg	116.1 ± 75.8	105.5 ± 59.6	0.4798
PaCO_2_, mmHg	39.0 ± 14.3	43.5 ± 19.2	0.2096
PaO_2_/FiO_2_ ratio	237.49 ± 130.11	222.37 ± 139.45	0.6021
Na, mmol/L	139.7 ± 7.5	141.0 ± 6.7	0.4019
K, mmol/L	4.2 ± 0.9	4.1 ± 0.8	0.6041
Ca, mmol/L	2.1 ± 0.2	2.0 ± 0.2	0.0033
Mg, mmol/L	1.0 ± 0.2	1.0 ± 0.1	0.3299
P, mmol/L	1.2 ± 0.3	1.3 ± 0.2	0.0727
Albumin, g/L	30.4 ± 3.1	27.6 ± 4.3	0.0007
Hemoglobin, g/L	97.7 ± 18.5	86.9 ± 12.7	0.0028
White blood cell, × 10^9^/L	14.3 ± 7.5	14.0 ± 11.3	0.8970
Creatinine, μmol/L	198.7 ± 205.0	258.2 ± 238.3	0.2123
NT-proBNP*, pg/ml	2436.00 (465.25–12,375.00)	2994.50 (1841.50–5534.00)	0.9882
APHACHE II	23.3 ± 7.7	24.3 ± 7.3	0.5239
SOFA score	4.4 ± 1.6	5.6 ± 2.3	0.0054
*Comorbidity*			
Hypertension, n (%)	19, 38.0%	14, 36.8%	0.9115
Diabetes mellitus, n (%)	7, 14.0%	8, 21.1%	0.3835
Coronary heart disease, n (%)	3, 6.0%	15, 39.5%	0.0001
Chronic kidney disease, n (%)	6, 12.0%	11, 29.0%	0.0461
COPD, n (%)	10, 20.0%	12, 31.6%	0.2140
Tumor, n (%)	2, 4.0%	6, 15.8%	0.1257
Sepsis, n (%)	21, 42.0%	24, 63.2%	0.0492

*PEEP* positive end-expiratory pressure; *APHACHE* Acute Physiology and Chronic Health Evaluation; *SOFA* Sequential Organ Failure Assessment; *NT-proBNP* N-terminal pro-B type natriuretic peptide; *COPD* chronic obstructive pulmonary disease

*Result of NT-proBNP was expressed as median (quantile)

The mean, maximal, and minimal excursion of the diaphragm and velocity measured by speckle tracking imaging and manual assessment are listed in Table 2. The mean automatic diaphragmatic excursion was significantly lower among patients of the weaning-failure group, as compared with the weaning-success group (1.1 vs. 1.5 cm, *p* = 0.0163). Diaphragmatic velocity of excursion (cm/s) were similar among patients of both group (1.0 vs. 0.9, *p* = 0.2437). Mean diaphragmatic excursion via manual imaging assessment was 0.9 ± 0.4 cm in the weaning-success group and 0.9 ± 0.7 cm in the weaning-failure group (*p* = 0.6681). The mean diaphragmatic velocity of excursion via manual imaging assessment was 1.4 ± 0.7 cm/s in the weaning-success group compared with 1.4 ± 0.8 in the weaning-failure group, with no significant differences between groups. (Table 2).

**Table 2:** Ultrasonic variables of automatic speckle tracking and manual measurement between weaning-failure and weaning-success groups

	Weaning-success *N* = 50	Weaning-failure *N* = 38	*p* value
*Automatic measurement*			
Mean excursion, cm	1.5 ± 0.8	1.1 ± 0.6	0.0163
Max excursion, cm	1.6 ± 0.9	1.2 ± 0.6	0.0143
Min excursion, cm	1.3 ± 0.8	1.0 ± 0.6	0.0270
*Manual measurement*			
Excursion, cm	0.9 ± 0.4	0.9 ± 0.7	0.6681
*Automatic measurement*			
Max velocity, cm/s	1.2 ± 0.7	1.0 ± 0.6	0.1837
Min velocity, cm/s	0.9 ± 0.7	0.8 ± 0.5	0.2747
Mean velocity, cm/s	1.0 ± 0.7	0.9 ± 0.5	0.2437
*Manual measurement*			
Velocity, cm/s	1.4 ± 0.7	1.4 ± 0.8	0.8011

**Fig. 2:**
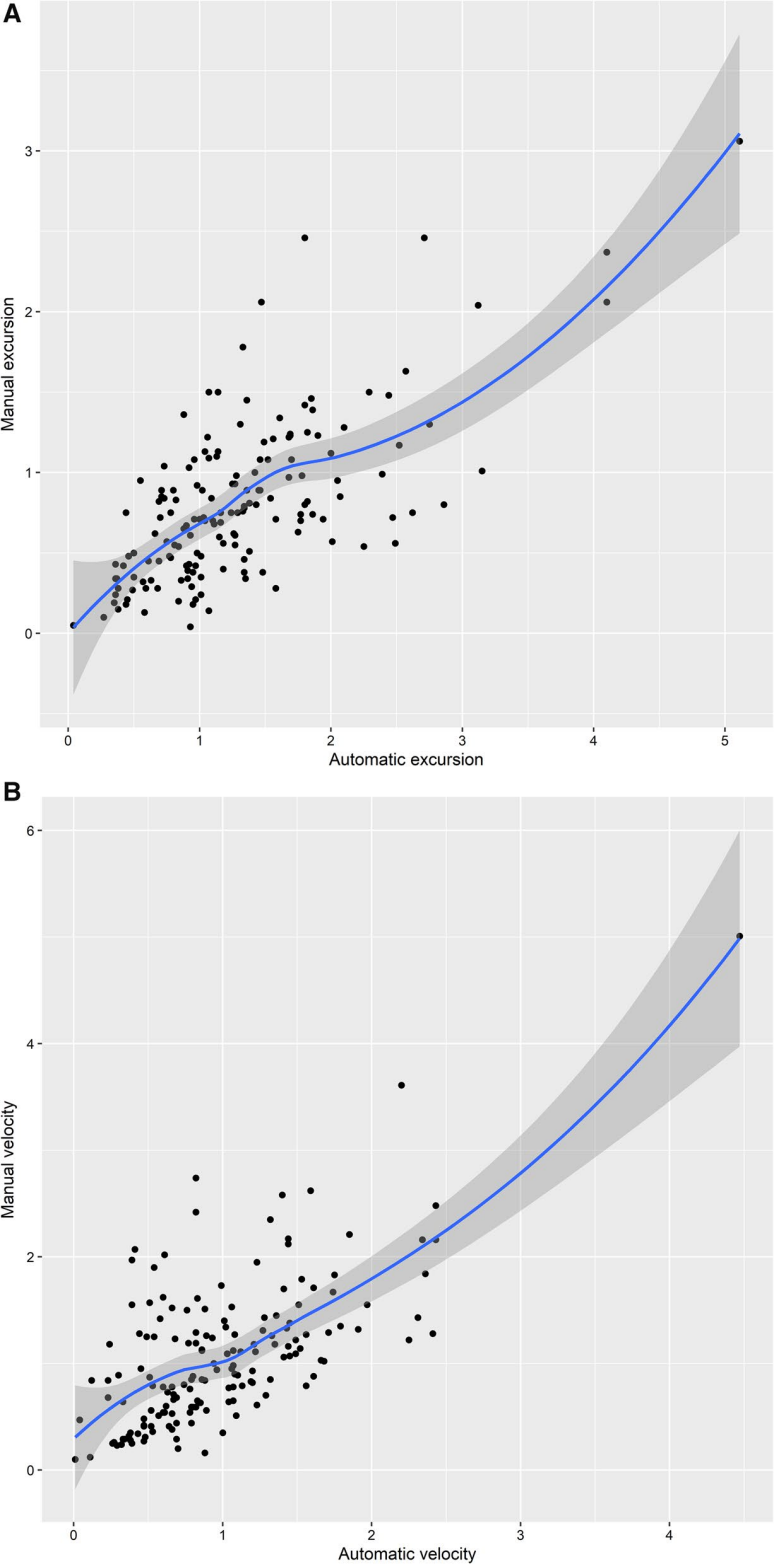
**A** Scatter Plot and Fitting Curve of Average Excursion of Automatic Measurement and Manual Measurement (R 0.69). **B** Scatter Plot and Fitting Curve of Average Velocity of Automatic Measurement and Manual Measurement (R 0.65)

Excursion and velocity were both significantly correlated with manual measurement. There was a significant correlation between the automatic measurement of mean excursion assessed by speckle tracking imaging and manual measurement (R 0.69, *p* < 0.0001, Fig. 2A), while the correlation between automatic velocity and manual data was also observed (R 0.65, *p* < 0.0001, Fig. 2B). Bland–Altman plot showed the representations of the agreement between manual and automatic measurements of diaphragmatic excursion (bias: − 0.5 cm; LOA: − 1.6–0.6 cm, Additional file 5: Fig S1) and velocity (bias: 0.1 cm/s; LOA: − 1.0–1.2 cm/s, Additional file 6: Fig. S2). Additional file 7: Table S1 shows the ultrasonic variables between left and right diaphragm.

**Fig. 3:**
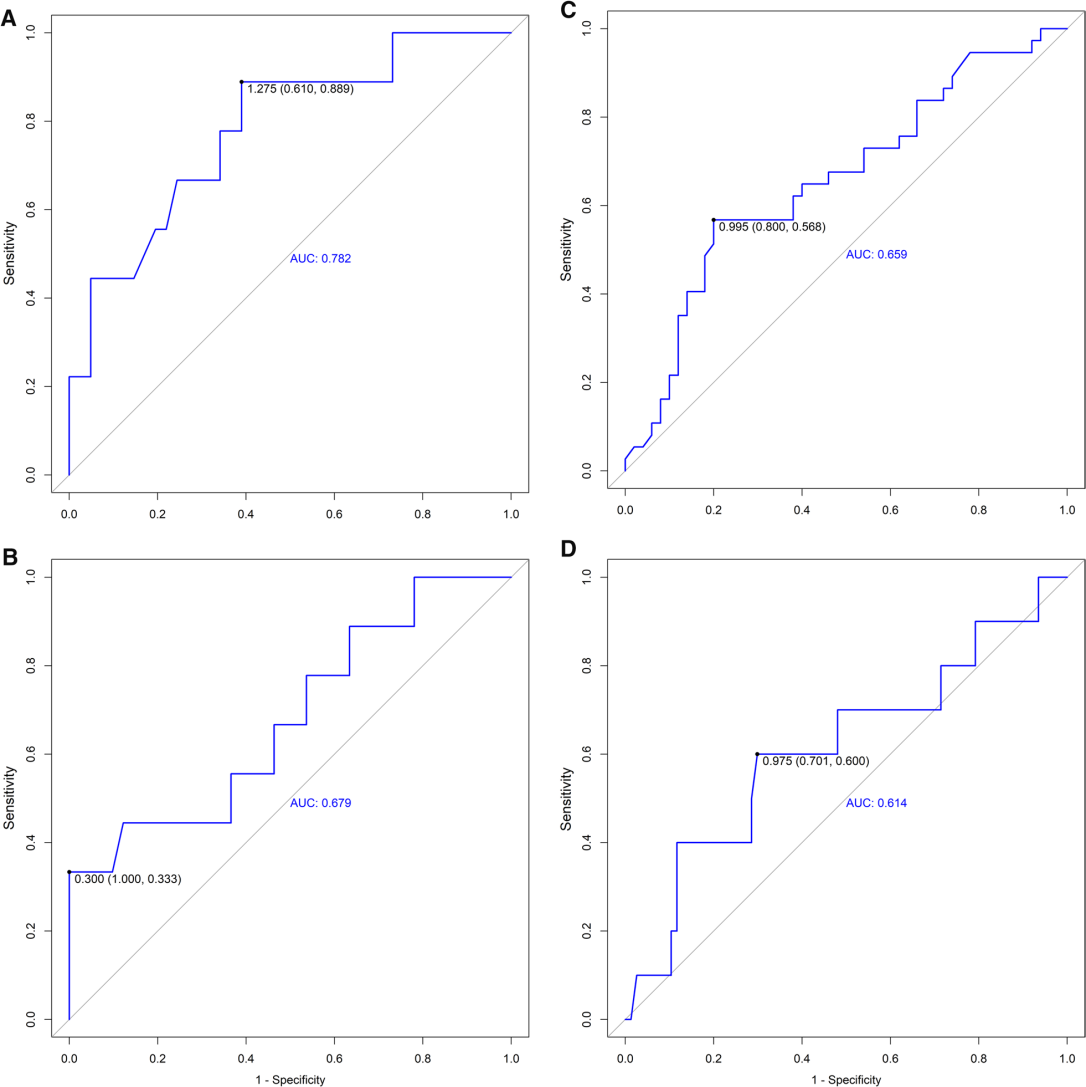
**A** ROC Curve Shows the Diagnostic Value for Prolonged Weaning of the Automatic Mean Excursion (Cut-Off 1.275, AUROC 0.782). **B** ROC Curve Shows the Diagnostic Value for Prolonged Weaning of the Automatic Mean Velocity (Cut-Off 0.300, AUROC 0.679). **C** ROC Curve Shows the Diagnostic Value for Weaning-Failure of the Automatic Mean Excursion (Cut-Off 0.995, AUROC 0.659). **D** Curve Shows the Diagnostic Value for In-hospital Death/Withdrawal of treatment of the Automatic Mean Excursion (Cut-Off 0.975, AUROC 0.614)

The best cut-off values and area under the curve for weaning failure were calculated for automatic diaphragmatic excursion and diaphragmatic velocity. We performed a ROC curve analysis to assess the predictive value of these variables. The mean diaphragmatic excursion exhibited high diagnostic values for prolonged weaning (Fig. 3A) with the area under the receiver operating characteristic curve (AUROC) of 0.782. The sensitivity and specificity for predicting prolonged weaning were 88.9% and 61.0%. The mean diaphragmatic velocity of excursion also exhibited moderate diagnostic values for prolonged weaning (Fig. 3B) with an AUROC of 0.679. The sensitivity and specificity for predicting prolonged weaning were 33.3% and 100%. Besides, the diaphragmatic excursion showed moderate diagnostic value for predicting both weaning failure (Fig. 3C) and in-hospital death/withdrawal of treatment (Fig. 3D).

## Discussion

The diaphragm plays a significant role in ventilation, and its dysfunction can result in difficulty weaning from MV [4]. The diaphragm weakness developed rapidly in the first few days of MV [12–14]. Growing evidence showed that diaphragm dysfunction contributed to weaning failure and prolonged ventilation [11, 13, 15, 16]. Diaphragmatic function assessment is vital in critically ill patients and this part of the population with invasive MV. It is also important to consider the factors that affect and predict the success of ventilator weaning.

Diaphragm ultrasound can currently be performed at the bedside to monitor the diaphragm movement, the diaphragm thickness, and the thickening rate [8, 17, 18]. However, conventional methods, such as thickness fraction, or caudal displacement assessed by M-mode have limitations, such as angle dependence and translational error. At present, there is no standard quantification for measuring diaphragmatic movement, because the moderate consistency of two-dimensional ultrasound was a bottleneck problem. Our study aimed to provide a novel quantifiable method of diaphragm function analysis.

In this study, the speckle tracking technology was used to measure the diaphragmatic excursion and its velocity. Compared to conventional ultrasound, it automatically chose three to six ROI on the diaphragm when measuring, calculated its speed or displacement in different parts of the diaphragm, and then form a general parameter, which was accurate and comprehensive to evaluate the excursion speed of the entire diaphragm. In addition, the original intention of developing the software was to standardize the measurement of the diaphragm ultrasound, including excursion and velocity.

Pesero et al. discovered the anatomical M-mode which allowed free placement of the cursor to measure diaphragmatic excursion and helped recognize diaphragmatic dysfunction since the conventional analysis line overestimated excursion in cardiac surgical patients[19]. Orde et al. proposed the use of angle-independent M-mode sonography for the assessment of diaphragm displacement, demonstrating that the cursor might not be orientated to the true direction of the diaphragm movement, leading to orientation and translation error[20]. Inspired by the previous studies, we calculated a calibration line during the automatic measurement (Additional file 8 videos 3 and Additional file 9: video 4). Our results suggested that the automatic measurement of diaphragmatic excursion velocity was lower than that obtained by manual measurement, which might be due to the use of the anatomic M-line adjusted algorithm. The abovementioned study suggested that the diaphragmatic excursion measured by conventional M-mode was overestimated [19]. It might partially explain the result of the automatic measurement of velocity. However, it should be emphasized that this was a newly developed software, which still needed to be trained with large sample data to achieve continuous improvement. Overall, the present study exhibited a scenario where diaphragmatic kinetics assessment could be performed via automatic measurement.

In addition, the low excursion and velocity might be contributed to the timing of the ultrasound. Ultrasound was performed before SBT in the present study while the previous literature reported ultrasound data collected during the first 30 min of SBT [11]. Cammarota et al. investigated the diaphragmatic excursion velocity measured with tissue Doppler imaging at the end of the SBT [21]. The result suggested that subjects who developed both extubation failure and success experienced a greater diaphragmatic activation, compared with the result in the present study. Upon MV assistance, diaphragmatic movement and contraction might not require too much effort due to the positive pressure support. Another study indicated that the mode of ventilation affected the preservation of diaphragmatic contraction, as MV support, could partially reverse the muscle atrophy process [22]. It might be mutually verified that the diaphragmatic excursion and velocity were affected by MV. For acutely hospitalized patients ventilated more than 24 h, guideline suggested that the initial SBT be conducted with inspiratory pressure augmentation (5–8 cm H_2_O) rather than without [23]. Using low-level pressure support or continuous positive airway pressure counteracted the resistance of the breathing circuit. The initial purpose of the diaphragmatic assessment was to predict the extubation success so that to avoid the potentially hazardous effects, such as respiratory muscle fatigue or dyspnea, caused by SBT. Therefore, we chose to perform the diaphragmatic assessment before SBT. Moreover, all of the patients had spontaneous breathing with the pressure support of 10–12 cm H_2_O. We believed that it simulated SBT with inspiratory pressure augmentation, to a certain extent.

Expert consensus recommended diaphragmatic movement ultrasound measurement and emphasized the importance of context-specific or outcome-related cut-off values[1]. The results of the present study showed that the correlation between manual measurement and automatic speckle tracking measurement was high. Follow-up data on clinical adverse outcomes were collected to validate the prognostic value. In the present study, a 43.18% (38/88 patients) incidence of weaning failure was observed. ROC curve analysis showed that a mean excursion ≤ 1.3 cm, and a mean velocity ≤ 0.3 cm/s represent possible predictors for prolonged weaning. The AUROC curves for these variables were 0.782 and 0.679, respectively, our results also suggested that a mean excursion ≤ 1.0 cm was predictive of weaning failure (AUROC = 0.659), while a mean excursion ≤ 1.0 cm prognosticated in-hospital death/withdrawal of treatment (AUROC = 0.614). The cut-off value was consistent with the diagnostic criteria of diaphragmatic dysfunction[24].

The software calculations are based on an algorithm patented, which is not open to the public. The present study provided a pilot vision toward a novel measurement for diaphragm ultrasonography in research and daily practice, compared with the currently used techniques.

There are limitations to the present study. Primarily, given the small sample size in our pilot study, a larger, multicentered study could be useful to validate the role of the current software module. Second, speckle tracking could be applied to automatically measure the thickness and changing rate of the diaphragm. The rate of change in the thickness of the diaphragm, plus the excursion and velocity data that has been achieved so far, may be valuable in the evaluation of the diaphragm function. A combination of several parameters might provide multiple dimensions and enhance predictive power.

## Conclusion

Automatic speckle tracking analysis of the diaphragm showed high consistency with conventional manual ultrasound measures. Diaphragmatic excursion and its velocity helped predict MV weaning failure, prolonged weaning, as well as in-hospital adverse outcomes. The automatic speckle tracking ultrasound imaging module served as a reliable tool to predict weaning outcomes at the bedside, holding a promising prospect in guiding clinical weaning strategy.

### Supplementary Information

**Additional file 1**. Process of ultrasound imaging.

**Additional file 2**.** Video 1**. During the identification phase, speckle-tracking technology was used to develop an automatic measurement module for diaphragmatic ultrasound, to identify the diaphragm region through pattern recognition, deep learning, and other methods. The software selected 3 regions of interest (ROI) to represent each segment of the diaphragm (color red, yellow, and blue), and automatically measure the motion trajectory, speed, excursion, and other parameters from each ROI through a certain algorithm.

**Additional file 3**.** Video 2**. The recording clip of ultrasound images in both Video 2 and Video 1 was identical. The software selected 6 regions of interest (ROI) to represent each segment of the diaphragm (color red, yellow, blue, pink, grey, and green).

**Additional file 4**. Process of determining the anatomic M-line.

**Additional file 5**. **Figure S1**. Bland-Altman plot: representations of the agreement between manual and automatic measurement of diaphragmatic excursion

**Additional file 6**. **Figure S2**. Bland-Altman plot: representations of the agreement between manual and automatic measurement of diaphragmatic velocity.

**Additional file 7**. **Table S1**. Ultrasonic Variables of Automatic Speckle Tracking and Manual Measurement between left and right diaphragm.

**Additional file 8**.** Video 3**. During the validation phase. The software selected 3 regions of interest (ROI) to represent each segment of the diaphragm (color red). The line chart showed that the diaphragm excursion (cm), measured by one ROI, changed continuously along with the respiratory cycle, which was measured by ROI. The box in the upper left corner represented diaphragmatic excursion within every respiratory cycle (the former number was the frames in which the ultrasound image was acquired and the next number was excursion).

**Additional file 9**.** Video 4**. During the validation phase. The software selected 3 regions of interest (ROI) to represent each segment of the diaphragm (color red). The line chart showed that the diaphragm excursion (cm), measured by one ROI, changed continuously along with the respiratory cycle, which was measured by ROI. The box in the upper left corner represented diaphragmatic excursion within every respiratory cycle (the former number was the frames in which the ultrasound image was acquired and the next number was excursion).

## Data Availability

The data sets generated and/or analyzed during the current study are not publicly available to protect the privacy of participants but are available from the corresponding author upon reasonable request. The study protocol was approved by the ethics committee of Guangdong Provincial People’s Hospital/ Guangdong Academy of Medical Sciences (No. 2020-246H-1) and Guangzhou Panyu Central Hospital (No. PYRC-2021–115), and written informed consent was obtained from each patient’s family.

## References

[1] Haaksma ME, Smit JM, Boussuges A, Demoule A, Dres M, Ferrari G, Formenti P, Goligher EC, Heunks L, Lim EHT (2022). EXpert consensus on diaphragm ultrasonography in the critically ill (EXODUS): a Delphi consensus statement on the measurement of diaphragm ultrasound-derived parameters in a critical care setting. Crit Care.

[2] Zambon M, Greco M, Bocchino S, Cabrini L, Beccaria PF, Zangrillo A (2017). Assessment of diaphragmatic dysfunction in the critically ill patient with ultrasound: a systematic review. Intensive Care Med.

[3] Vetrugno L, Guadagnin GM, Barbariol F, Langiano N, Zangrillo A, Bove T (2019). Ultrasound imaging for diaphragm dysfunction: a narrative literature review. J Cardiothorac Vasc Anesth.

[4] Kim WY, Suh HJ, Hong SB, Koh Y, Lim CM (2011). Diaphragm dysfunction assessed by ultrasonography: influence on weaning from mechanical ventilation. Crit Care Med.

[5] Sarwal A, Walker FO, Cartwright MS (2013). Neuromuscular ultrasound for evaluation of the diaphragm. Muscle Nerve.

[6] McCool FD, Tzelepis GE (2012). Dysfunction of the diaphragm. N Engl J Med.

[7] Gerscovich EO, Cronan M, McGahan JP, Jain K, Jones CD, McDonald C (2001). Ultrasonographic evaluation of diaphragmatic motion. J Ultrasound Med.

[8] Boussuges A, Gole Y, Blanc P (2009). Diaphragmatic motion studied by m-mode ultrasonography: methods, reproducibility, and normal values. Chest.

[9] Amundsen BH, Helle-Valle T, Edvardsen T, Torp H, Crosby J, Lyseggen E, Stoylen A, Ihlen H, Lima JA, Smiseth OA (2006). Noninvasive myocardial strain measurement by speckle tracking echocardiography: validation against sonomicrometry and tagged magnetic resonance imaging. J Am Coll Cardiol.

[10] Rubin JM, Feng M, Hadley SW, Fowlkes JB, Hamilton JD (2012). Potential use of ultrasound speckle tracking for motion management during radiotherapy: preliminary report. J Ultrasound Med.

[11] Huang D, Ma H, Zhong W, Wang X, Wu Y, Qin T, Wang S, Tan N (2017). Using M-mode ultrasonography to assess diaphragm dysfunction and predict the success of mechanical ventilation weaning in elderly patients. J Thorac Dis.

[12] Hooijman PE, Beishuizen A, Witt CC, de Waard MC, Girbes AR, Spoelstra-de Man AM, Niessen HW, Manders E, van Hees HW, van den Brom CE (2015). Diaphragm muscle fiber weakness and ubiquitin-proteasome activation in critically ill patients. Am J Respir Crit Care Med.

[13] Jaber S, Petrof BJ, Jung B, Chanques G, Berthet JP, Rabuel C, Bouyabrine H, Courouble P, Koechlin-Ramonatxo C, Sebbane M (2011). Rapidly progressive diaphragmatic weakness and injury during mechanical ventilation in humans. Am J Respir Crit Care Med.

[14] Levine S, Nguyen T, Taylor N, Friscia ME, Budak MT, Rothenberg P, Zhu J, Sachdeva R, Sonnad S, Kaiser LR (2008). Rapid disuse atrophy of diaphragm fibers in mechanically ventilated humans. N Engl J Med.

[15] Hermans G, Agten A, Testelmans D, Decramer M, Gayan-Ramirez G (2010). Increased duration of mechanical ventilation is associated with decreased diaphragmatic force: a prospective observational study. Crit Care.

[16] Laghi F, Cattapan SE, Jubran A, Parthasarathy S, Warshawsky P, Choi YS, Tobin MJ (2003). Is weaning failure caused by low-frequency fatigue of the diaphragm?. Am J Respir Crit Care Med.

[17] Noh DK, Lee JJ, You JH (2014). Diaphragm breathing movement measurement using ultrasound and radiographic imaging: a concurrent validity. Biomed Mater Eng.

[18] Rocco M, Carbone I, Morelli A, Bertoletti L, Rossi S, Vitale M, Montini L, Passariello R, Pietropaoli P (2008). Diagnostic accuracy of bedside ultrasonography in the ICU: feasibility of detecting pulmonary effusion and lung contusion in patients on respiratory support after severe blunt thoracic trauma. Acta Anaesthesiol Scand.

[19] Pasero D, Koeltz A, Placido R, Fontes Lima M, Haun O, Rienzo M, Marrache D, Pirracchio R, Safran D, Cholley B (2015). Improving ultrasonic measurement of diaphragmatic excursion after cardiac surgery using the anatomical M-mode: a randomized crossover study. Intensive Care Med.

[20] Orde SR, Boon AJ, Firth DG, Villarraga HR, Sekiguchi H (2016). Use of angle-independent M-mode sonography for assessment of diaphragm displacement. J Ultrasound Med.

[21] Cammarota G, Boniolo E, Santangelo E, De Vita N, Verdina F, Crudo S, Sguazzotti I, Perucca R, Messina A, Zanoni M (2021). Diaphragmatic kinetics assessment by tissue doppler imaging and extubation outcome. Respir Care.

[22] Grassi A, Ferlicca D, Lupieri E, Calcinati S, Francesconi S, Sala V, Ormas V, Chiodaroli E, Abbruzzese C, Curto F (2020). Assisted mechanical ventilation promotes recovery of diaphragmatic thickness in critically ill patients: a prospective observational study. Crit Care.

[23] Schmidt GA, Girard TD, Kress JP, Morris PE, Ouellette DR, Alhazzani W, Burns SM, Epstein SK, Esteban A, Fan E (2017). Liberation from mechanical ventilation in critically ill adults: executive summary of an Official American College of Chest Physicians/American Thoracic Society clinical practice guideline. Chest.

[24] Umbrello M, Formenti P, Longhi D, Galimberti A, Piva I, Pezzi A, Mistraletti G, Marini JJ, Iapichino G (2015). Diaphragm ultrasound as indicator of respiratory effort in critically ill patients undergoing assisted mechanical ventilation: a pilot clinical study. Crit Care.

