# Supplementary material for: Using automatic speckle tracking imaging to measure diaphragm excursion and predict the outcome of mechanical ventilation weaning
Source: Crit Care. 2023 Jan 14;27:18. doi: 10.1186/s13054-022-04288-3 (PMC9840291; doi:10.1186/s13054-022-04288-3)
Supplement: Supplementary file 1 — Additional file 1. Process of ultrasound imaging. [file 13054_2022_4288_MOESM1_ESM.docx]

Additional file 1

Title: Using Automatic Speckle Tracking Imaging to Measure Diaphragm Excursion and Predict the Outcome of Mechanical Ventilation Weaning

Authors: Daozheng Huang, Feier Song, Bangjun Luo, Shouhong Wang, Tiehe Qin, Zhuandi Lin, Tieying Hou, Huan Ma

ultrasound machine: TE7 Diagnostic Ultrasound System (C5-2 array probe, Shenzhen Mindray Bio-medical, China)

Process of ultrasound imaging via convex array probe

1. input demographics and record clinical characteristics: patient’s ID, name, age, and gender. Data on MV parameters were collected.

2. 30° head-up (or supine or semi-recumbent position according to clinical practice).

3. Connect the convex array probe (default is diaphragm mode).

4. Data collection.

Data collected from the right diaphragm: the liver was used as the acoustic window to ensure that the diaphragm was as complete and clear as possible and in the center of the display interface. Image acquisition follows one of the following methods.

(1) scan in the longitudinal or transverse direction under the costal margin of the midclavicular line.

(2) scan in the longitudinal or transverse direction through the anterior axillary line.

(3) scan in the longitudinal or transverse direction through the posterior axillary line.

A sequence of images to cover 3-5 respiratory cycle was obtained. After collection, click “Annotate” to input right diaphragm annotation (RT), and then click “keyboard” to record the image acquisition locations, including midclavicular line (SZ); anterior axillary line (YQ), posterior axillary line (YH), and transverse (H) or longitudinal (Z) section.

Data collected from the left diaphragm: the spleen was used as the acoustic window to ensure that the diaphragm was as complete and clear as possible and in the center of the display interface. Image acquisition follows the following two methods.

(1) scan in the longitudinal or transverse direction through the anterior axillary line.

(2) scan in the longitudinal or transverse direction through the posterior axillary line.

A sequence of images to cover 3-5 respiratory cycle was obtained. After collection, click “Annotate” to input left diaphragm annotation (LT), and then click “keyboard” to record the image acquisition locations, including anterior axillary line (YQ), posterior axillary line (YH), and transverse (H) or longitudinal (Z) section.

5. Click “save cline” to save the prior video, with images taken before the transducer was placed and while attempting for the optimal images. Record 3 respiratory cycles once the image stabilizes.
