# Supplementary material for: Using automatic speckle tracking imaging to measure diaphragm excursion and predict the outcome of mechanical ventilation weaning
Source: Crit Care. 2023 Jan 14;27:18. doi: 10.1186/s13054-022-04288-3 (PMC9840291; doi:10.1186/s13054-022-04288-3)
Supplement: Supplementary file 4 — Additional file 4. Process of determining the anatomic M-line. [file 13054_2022_4288_MOESM4_ESM.docx]

Additional file 4

Title: Using Automatic Speckle Tracking Imaging to Measure Diaphragm Excursion and Predict the Outcome of Mechanical Ventilation Weaning

Authors: Daozheng Huang, Feier Song, Bangjun Luo, Shouhong Wang, Tiehe Qin, Zhuandi Lin, Tieying Hou, Huan Ma

Process of determining the anatomic M-line

Definition: The dashed line in the supplement videos 3& 4 was the anatomical M-line, which was defined as the direction of the first complete maximum excursion of the diaphragm, namely the direction from the lowest (start) to the highest point (endpoint) of diaphragm movement. The excursion of the ROI on the diaphragm calculated by the algorithm was the projection of the excursion between the start and the endpoint on the anatomical M-line.

Steps to draw the anatomic M-line: (take the middle ROI as an example midpoint)

(1) Select 2 equidistant points from the midpoint on the diaphragm (Figure 3, color green point A and color black point B).

(2) In the first frame, calculate the tangent vector of the midpoint (direction vector of points A and B), as shown in the blue line.

(3) Calculate the normal vector of the midpoint (perpendicular to the tangent vector), as shown in the yellow line.


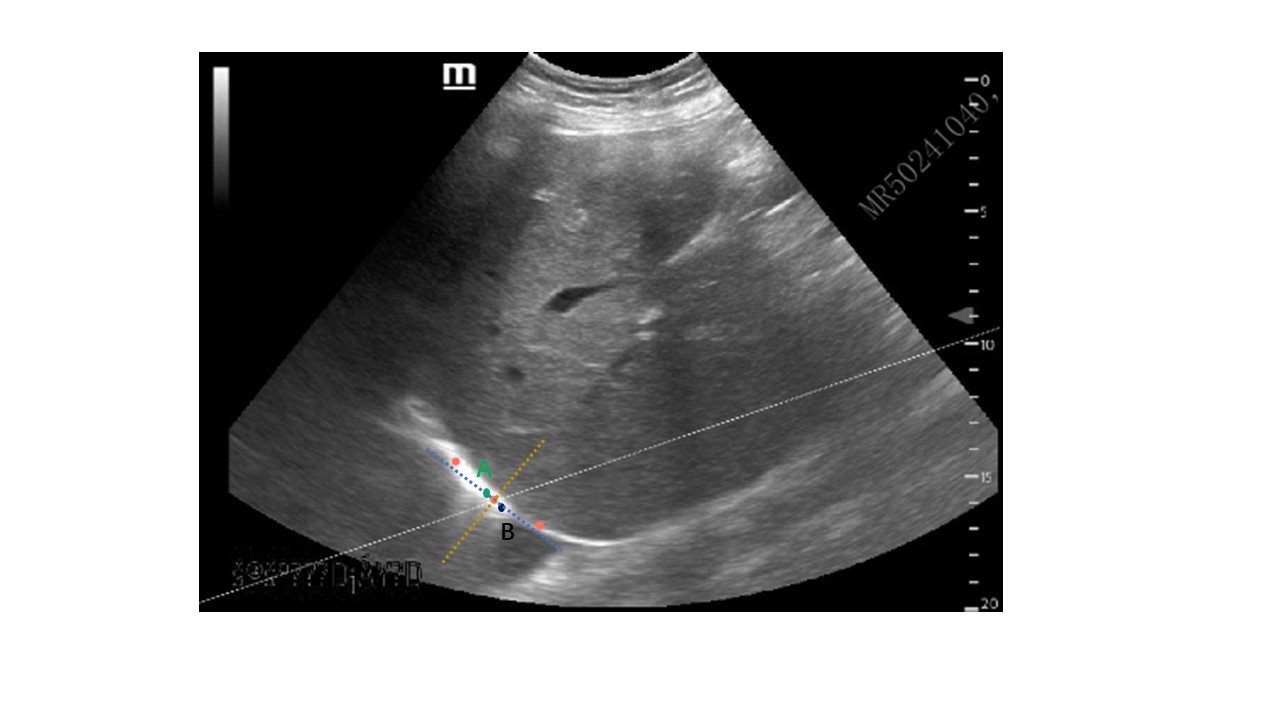


Figure 3

(4) Obtain the position (coordinate) of the midpoint in each frame of the video.

(5) The speed of the first frame was calculated:

the excursion (of the midpoint)/the interval of the two frames.

(6) Calculate the projection velocity of the midpoint of each frame on the tangent vector and normal vector.

(7) When the velocity directions of the tangent vector and normal vector both changed to the opposite, the start or the endpoint was identified. A complete excursion was recognized where the velocity direction successively changed.

(8) The anatomic M-line was drawn via calculation by the coordinates of the start and endpoint of the first excursion (Figure 3, dashed line).
