# Supplementary figures and images for: Using automatic speckle tracking imaging to measure diaphragm excursion and predict the outcome of mechanical ventilation weaning
Source: Crit Care. 2023 Jan 14;27:18. doi: 10.1186/s13054-022-04288-3 (PMC9840291; doi:10.1186/s13054-022-04288-3)

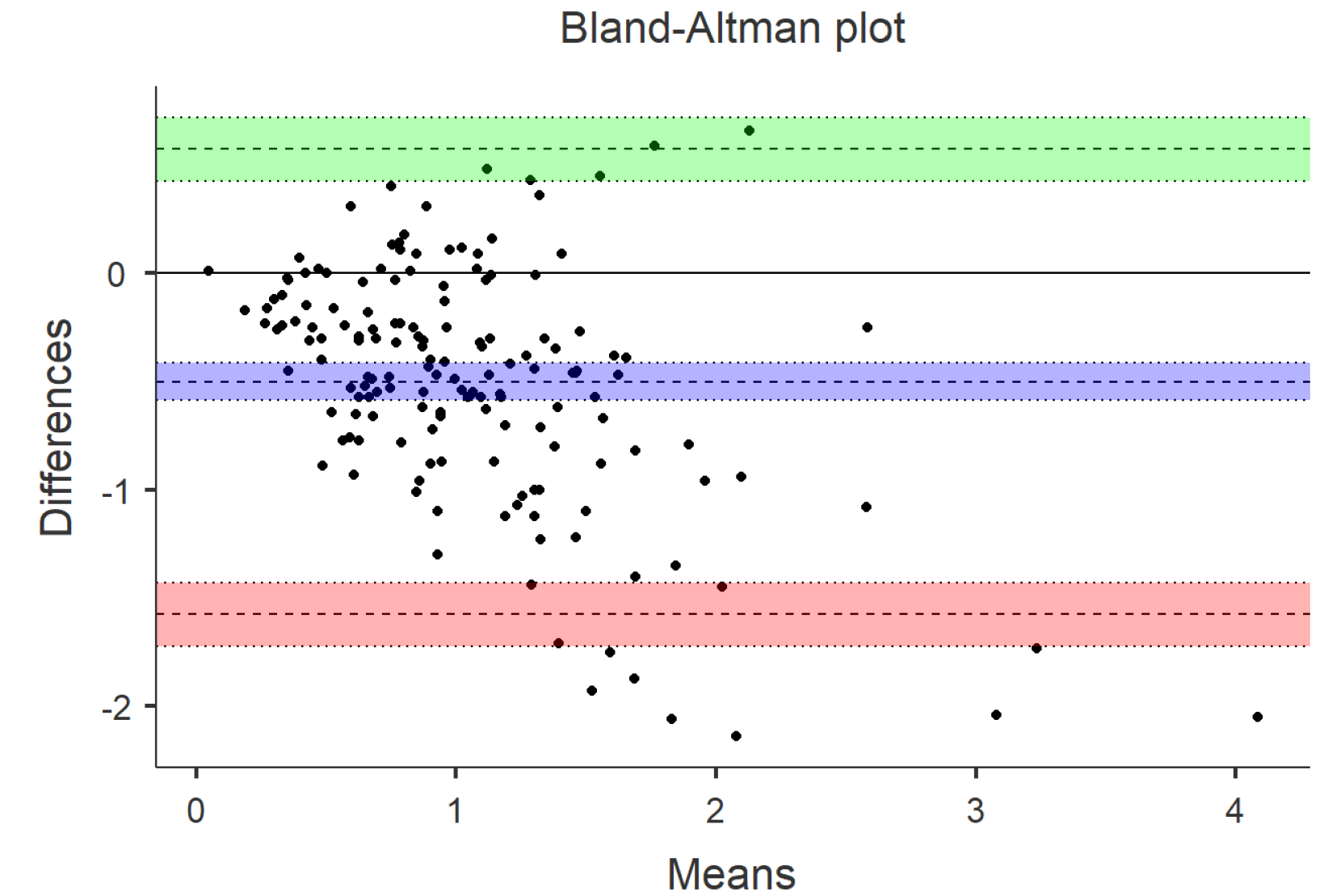

Supplement: Supplementary file 5 — Additional file 5. Figure S1. Bland-Altman plot: representations of the agreement between manual and automatic measurement of diaphragmatic excursion [file 13054_2022_4288_MOESM5_ESM.png]

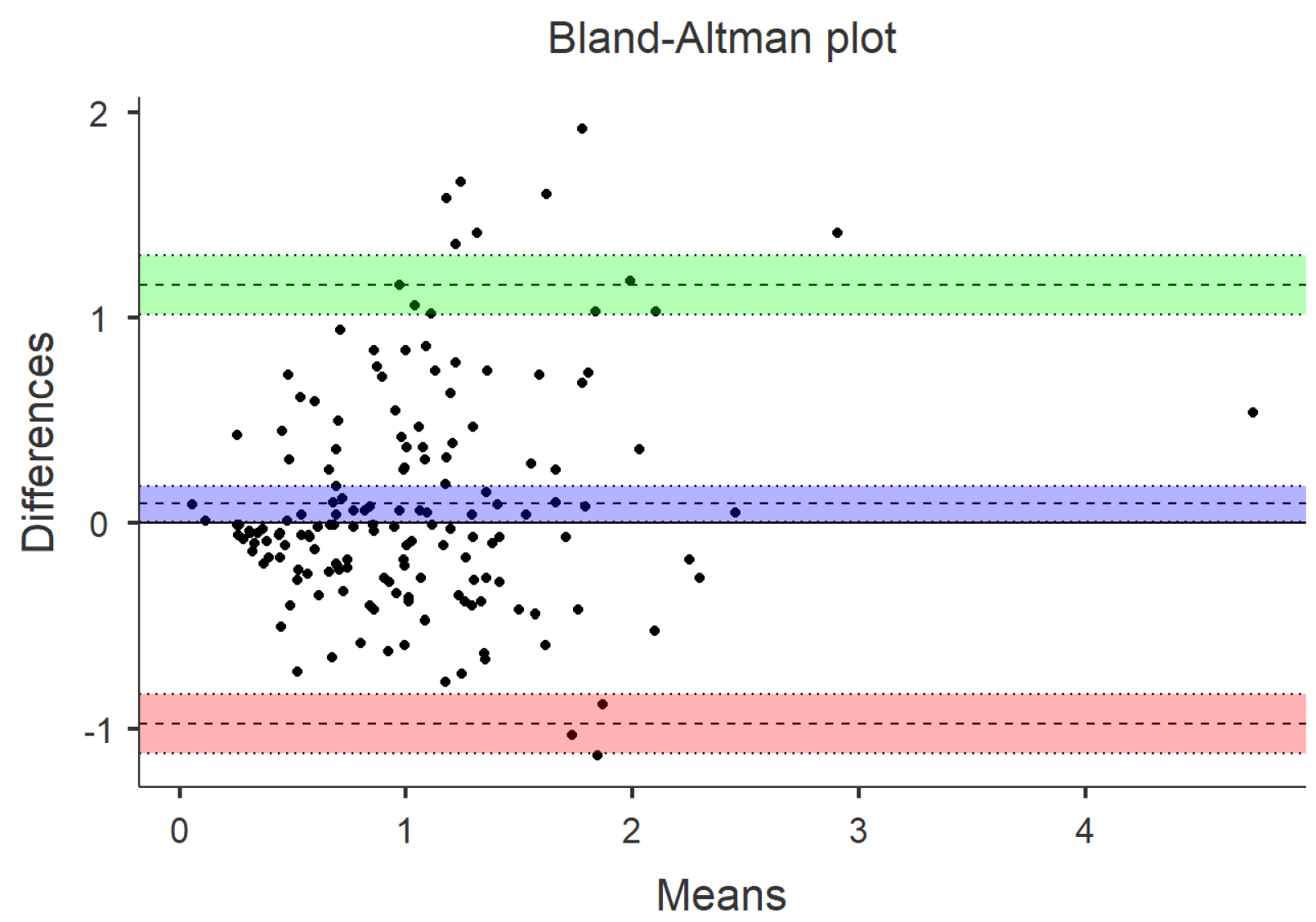

Supplement: Supplementary file 6 — Additional file 6. Figure S2. Bland-Altman plot: representations of the agreement between manual and automatic measurement of diaphragmatic velocity. [file 13054_2022_4288_MOESM6_ESM.png]
