# Supplementary material for: Using automatic speckle tracking imaging to measure diaphragm excursion and predict the outcome of mechanical ventilation weaning
Source: Crit Care. 2023 Jan 14;27:18. doi: 10.1186/s13054-022-04288-3 (PMC9840291; doi:10.1186/s13054-022-04288-3)
Supplement: Supplementary file 7 — Additional file 7. Table S1. Ultrasonic Variables of Automatic Speckle Tracking and Manual Measurement between left and right diaphragm. [file 13054_2022_4288_MOESM7_ESM.docx]

Table S1 Ultrasonic Variables of Automatic Speckle Tracking and Manual Measurement between left and right diaphragm

|  | Left | Right |
| --- | --- | --- |
| Automatic Measurement |  |  |
| Mean excursion, cm | 1.2±0.7 | 1.3±0.8 |
| Max excursion, cm | 1.3±0.8 | 1.5±0.8 |
| Min excursion, cm | 1.1±0.7 | 1.2±0.7 |
| Manual Measurement |  |  |
| Excursion, cm | 0.8±0.5 | 0.8±0.5 |
| Automatic Measurement |  |  |
| Max velocity, cm/s | 1.0±0.5 | 1.1±0.7 |
| Min velocity, cm/s | 0.8±0.5 | 0.9±0.6 |
| Mean velocity, cm/s | 0.9±0.5 | 1.0±0.6 |
| Manual Measurement |  |  |
| Velocity, cm/s | 1.0±0.6 | 1.1±0.7 |
